# A Locomotion Intent Prediction System Based on Multi-Sensor Fusion

**DOI:** 10.3390/s140712349

**Published:** 2014-07-10

**Authors:** Baojun Chen, Enhao Zheng, Qining Wang

**Affiliations:** Intelligent Control Laboratory, College of Engineering, Peking University, Beijing 100871, China; E-Mails: chenbaojun@pku.edu.cn (B.C.); zhengenhao@pku.edu.cn (E.Z.)

**Keywords:** locomotion mode recognition, locomotion transition detection, sensor fusion, linear discriminant analysis, lower-limb prosthesis

## Abstract

Locomotion intent prediction is essential for the control of powered lower-limb prostheses to realize smooth locomotion transitions. In this research, we develop a multi-sensor fusion based locomotion intent prediction system, which can recognize current locomotion mode and detect locomotion transitions in advance. Seven able-bodied subjects were recruited for this research. Signals from two foot pressure insoles and three inertial measurement units (one on the thigh, one on the shank and the other on the foot) are measured. A two-level recognition strategy is used for the recognition with linear discriminate classifier. Six kinds of locomotion modes and ten kinds of locomotion transitions are tested in this study. Recognition accuracy during steady locomotion periods (*i.e.*, no locomotion transitions) is 99.71% ± 0.05% for seven able-bodied subjects. During locomotion transition periods, all the transitions are correctly detected and most of them can be detected before transiting to new locomotion modes. No significant deterioration in recognition performance is observed in the following five hours after the system is trained, and small number of experiment trials are required to train reliable classifiers.

## 1. Introduction

Lower-limb prostheses greatly improve amputees' locomotive ability in performing daily activities. Most of the commercially available lower-limb prostheses are energetically passive. Though they are widely used, they have inevitable deficiencies such as more metabolic power consumption and asymmetrical gait pattern [1]. Thus, studies on powered lower-limb prostheses are gaining more and more attention [1–6]. Compared with passive lower-limb prostheses, powered prostheses can provide necessary power in certain periods to make the locomotion more natural, but they need more complex control strategies [7–12] and more locomotion information from amputees. As control approaches for different locomotion modes are different, powered prostheses should “know” amputees' locomotion intents in advance, so as to select appropriate parameters and realize safe and fluent control. Therefore, the prediction of amputees' locomotion intents plays an important role for the control of powered lower-limb prostheses.

Some methods have been proposed for lower-limb locomotion mode recognition using signals measured from mechanical sensors [13–15] and bioelectric signals such as electromyography (EMG) signals [16–20]. Varol *et al.* collected signals of joint angles and angular velocities of the knee and ankle, socket sagittal plane moment, foot forces of heel and ball, and realized the recognition of three locomotion modes (standing, sitting and walking) and transitions between them [14]. However, half a second delay existed for locomotion intent recognition. Young *et al.* proposed a recognition system capable of performing transitions between walking on level ground, ramps and stairs for a powered prosthesis, using onboard mechanical sensors [15]. However, the overall recognition accuracy was only 93.9%. Huang *et al.* used EMG signals measured from lower-limb and signals of a 6-DOF load cell to classify six locomotion modes and five locomotion transitions [18]. However, the method was only tested offline, and recognition performance of long-time use was not reported. The evaluation of long-time use is important for myoelectric decoding algorithms, because they are inherently subject to decay in recognition accuracy over time [21], which is caused by the variation of EMG signals due to electrode conductivity changes, electrophysiological changes, spatial changes, user changes and potentially other factors [22].

In principle, fusion of multi-sensor data provides significant advantages over single source data [23]. Therefore, sensor fusion is widely used in various fields such as robotics [24,25], medical applications [26], target tracking [27–29], person identity verification [30], human activity recognition [31,32] and so on. More and more intent prediction systems for powered lower-limb prosthesis control also developed with multi-sensor fusion [14,18]. Single kind of sensors only collect part of the locomotion information, which may be insufficient. With the fusion of different kinds of sensor signals, more useful information are obtained and recognition performance can therefore be improved. Huang *et al.* compared the recognition result of using only EMG signals with that using EMG-mechanical fusion, and validated that the performance with multi-sensor fusion was significantly better [18].

In this paper, we propose a locomotion intent prediction system based on a new way of multi-sensor fusion, and systematically evaluate its promise for clinical application. Signals of three inertial measurement units (IMUs) and two custom made pressure insoles are measured. These two kinds of sensors have been widely used for human movement measurement and detection [33–35]. Many works have been done on signal processing technologies for these sensors, and some methods have been proposed to deal with signal drift and interference [36]. In this research, with linear discriminant analysis (LDA) classifier, time-domain feature set and a two-level recognition strategy, the system can successfully recognize six locomotion modes and ten locomotion transitions. We also evaluated the performance of long-time use for this system on seven able-bodied subjects, which was essential for clinical application. No deteriorating tendency of recognition performance was observed after long-term use, and only a small data amount was needed for system training. These properties demonstrate the potential of applying the system for the control of powered lower-limb prostheses in daily life.

## 2. Measurement System

### 2.1. Sensor Placement on Human Body

**Figure 1.**
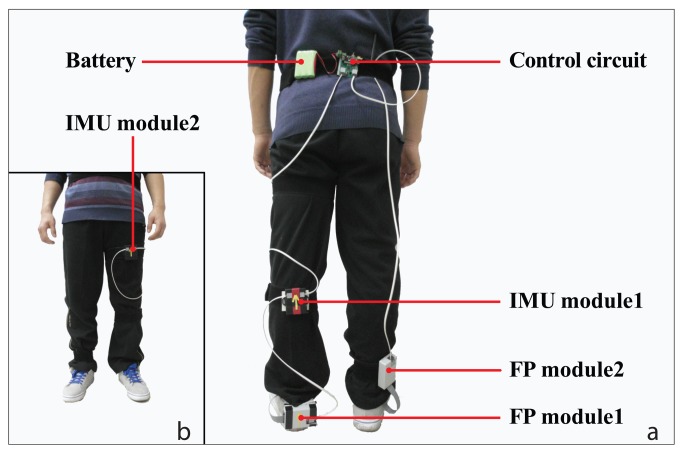
The placement of the sensors on human body. Foot pressure insoles are placed in shoes of both feet and the signals are sampled by circuits embedded in FP module1 and FP module2. IMUs used for recording the movement data of the thigh, the shank and the foot are embedded in IMU module1, IMU module2 and FP module1, respectively.

To measure as much useful locomotion information as possible, the positions of sensors were carefully selected (Figure 1). Three IMUs (SparkFun Electronics Inc., Boulder, CO, USA) were placed on the thigh (IMU module2), the shank (IMU module1), and the foot of the measured leg (FP module1), respectively. They were used to measure angles of the mentioned limbs in the sagittal plane and frontal plane, and accelerations along two perpendicular axes in the sagittal plane. In addition, two custom made pressure insoles were placed in shoes of both sides to detect gait events and record foot pressure information during stance periods. Foot pressure sampling circuits were placed on the shank for the unmeasured leg (FP module2) and the back of the shoe for the measured leg (FP module1). There was a control circuit on the waist to control the data sequence. A lithium battery (10.8 V) was used to provide power for the sensors.

### 2.2. Inertial Measurement Unit

**Figure 2.**
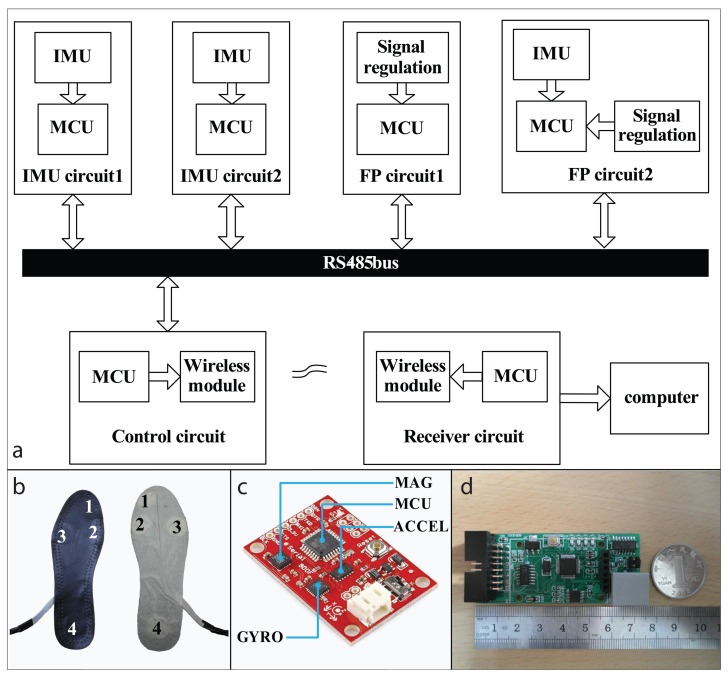
(**a**) The structure of data transmission in the designed system using RS-485 bus. In each sensor module, the data stream is controlled by MCU; (**b**) The custom made foot pressure insoles used in this study. Positions of pressure sensors are marked as 1, 2, 3 and 4, respectively; (**c**) The IMU board embedded with an accelerometer (ACCEL), a gyroscope (GYRO) and a magnetometer (MAG); (**d**) The foot pressure sampling circuit.

The IMU board is built with an accelerometer, a gyroscope and a magnetometer (Figure 2c). The accelerometer is a digital microchip named ADXL345 (Analog Devices Inc., Norwood, MA, USA) with three-axis measurement. The gyroscope is a three-axis micro-electromechanical systems (MEMS) based integrated circuit (ITG-3200, InvenSense Inc., San Jose, CA, USA) and the magnetometer is a surface-mount, multi-chip module designed for low-field magnetic sensing with a digital interface (HMC5883L, Honeywell International Inc., Morristown, NJ, USA). ATMEGA328 (Atmel Corp., San Jose, CA, USA) is used as the micro control unit (MCU) of the IMU board for data collection. The raw data measured with sensor chips are transmitted to the MCU via inter-integrated circuit (IIC) bus. To eliminate signal drift and noise, the direction-cosine-matrix (DCM) method is used. Data of the gyroscope are used for estimating the orientation of the IMU board. The magnetometer and the accelerometer are used to complement errors of the orientation. Output data of the IMU board include the pitch angle, the roll angle and accelerations in two axes, containing reliable and useful locomotion information.

### 2.3. Foot Pressure Insoles

The foot pressure insoles were self-made [37]. FlexiForce A401 (Tekscan Inc., South Boston, MA, USA) sensors were placed on the bottom of the insole to record foot pressure signals. The FlexiForce A401 sensor is a kind of force sensitive resistors which is inverse proportion to the force exerted on it. The sensors are selected for the following reasons. First, the resolution of the sensors is high enough to record the changes of foot pressure during stance periods. Second, the sensors are thin and small, making it comfortable to wear the equipped insoles. Third, the measuring range and the useful lifespan can meet the requirement for locomotion mode recognition. To collect more useful information of foot pressure during walking, positions of the sensors were carefully selected [37]. As is shown in Figure 2b, the four key positions are the big toe, the first metatarsal, the fourth metatarsal, and the heel. The position selection is similar to previous studies [38,39].

The foot pressure sampling circuits are designed to measure the foot pressure of both feet (Figure 2d). For each circuit, an inverting amplifier is built using LMV324 (STMicroelectronics Inc., Geneva, Switzerland) to convert the foot pressure to linear dependent voltage. Then the signals are input to the STM32F103 (STMicroelectronics Inc., Geneva, Switzerland) for analog to digital converting (ADC). For the sampling circuit on the measured leg, an IMU module is also embedded to measure movement information of the foot.

### 2.4. Data Transmission

The control circuit controls the data sequence of the sensor modules. In each sample interval, the control circuit collects data from each sensor module and then transmitted them to the receiver circuit with wireless module. The communication between the control circuit and the sensor modules is implemented with recommended standard 485 bus (Figure 2a). Sampling rates for all sensors are 100 Hz. In other words, all sensor data of each sample has to be sent to the receiver circuit in 10 ms. In the designed system, the data sequence is controlled with the polling method. In this method, each sensor module is assigned with an ID. Each sampling interval (10 ms) is separated to several time slices. During each slice, the control circuit broadcasts the command data packet with an ID and waits for the response. Then the sensor module with the same ID sends out the data. This method guarantees the unobstructed communication on the bus. The wireless module on the control circuit is built based on nRF24L01 (Nordic semiconductor Inc., Oslo, Norway). The wireless module sends the data out in each sample interval. Cyclic Redundancy Checking and automatic retransmission method is used to reduce error rate.

## 3. Methods

### 3.1. Subjects and Experiment Protocol

Seven able-bodied subjects participated in this research and provided written and informed consent. They had an average age of 24.1 (±0.5) years, an average height of 1.71 (±0.02) m and an average weight of 72.0 (±2.5) kg.

The experiment consisted of two sections. The first section included 20 experiment pairs. Each pair included one experiment trial with the measured leg first walking (marked as Set-A) and one trial with the unmeasured leg first walking (marked as Set-B). Experiment of the first section was taken continuously and five-minute rests were allowed every five pairs. The second section also included 20 experiment pairs. It started immediately after the first section. This section lasted for five hours and one experiment pair was tested every 15 min. In each experiment trial, different locomotion tasks were performed continuously in predefined order, so as to collect data of targeted locomotion transitions. The subjects stood still at the beginning, and then took level-ground walking, stair ascent, level-ground walking, ramp descent, level-ground walking, stand, turning back, stand, level-ground walking, ramp ascent, level-ground walking, stair descent, level-ground walking, stand, turning back and stand in turn. The staircase had 4 stairs and each one was 75 cm in width, 40 cm in depth and 15 cm in height. The inclination angle of the 2.1-meter long ramp was 16.5°.

Be similar with [14], the standing mode defined in this research included static standing (*i.e.*, standing still) and dynamic standing (turning back with small-step walking). For both static and dynamic standing, the powered prosthesis could work as a passive one because little energy was needed. In summary, six kinds of locomotion modes (level-ground walking (W), stair ascent (SA), stair descent (SD), ramp ascent (RA), ramp descent (RD) and standing (S)) and ten kinds of locomotion transitions (S→W, W→SA, SA→W, W→RD, RD→W, W→S, W→RA, RA→W, W→SD and SD→W) were tested. Note that transitions of S→W and W→S occurred twice in the experiment trial.

### 3.2. Classification

#### 3.2.1. Data for Classifier Training

As the measured leg played different roles in Set-A trials and Set-B trials, especially for locomotion transitions (in Set-A trials, the measured leg performed as the leading leg for transitions of S→W, W→SA, SA→W, W→RD, RD→W, and W→RA; in Set-B trials, the measured leg performed as the following leg for these transitions), there might be some differences between the data measured in the two kinds of experiment trials. In daily life, the leading leg for a locomotion transition is randomly selected (*i.e.*, either leg may perform as the leading leg). To make the classifier suitable for both situations, the data measured in Set-A and Set-B were used together for classifier training. The necessity of this training approach was verified by comparing recognition results with different combinations of training data and testing data.

#### 3.2.2. Data Segmentation and Labeling

To realize real-time locomotion mode recognition, we used overlapped sliding windows for data segmentation. In this case, some analysis windows contained data of two different modes when locomotion transitions occurred. If more than half of the analysis window was before the boundary of the two locomotion modes, the window was labeled as the former mode, otherwise it was labeled as the latter one. In this research, boundaries between two contiguous locomotion modes are defined as follows. For transitions from stand to other locomotion modes, the boundary was the moment when either foot left the ground. For transitions from other locomotion modes to stand, the boundary was the moment when the swing leg contacted the ground before standing still. For the rest locomotion transitions, the boundary was defined as the middle of swing period of the leg which first left the previous terrains. Window increment was 10 ms in this research.

The size of analysis window might be a factor influencing recognition performance. Larger analysis windows contained more information and might increase recognition accuracy, but also increased computation burden and might cause larger time delay for locomotion intent prediction. To systematically investigate the impact of window size on recognition, we varied window size from 100 ms to 200 ms and compared recognition performances.

#### 3.2.3. Two-Level Recognition Strategy

**Figure 3.**
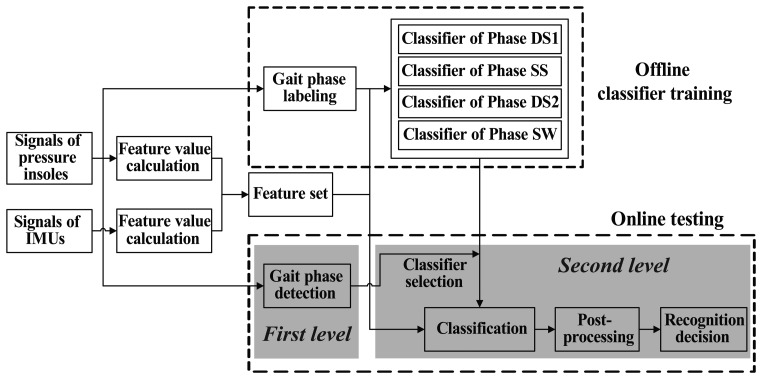
Block diagram of the locomotion intent prediction system.

Signals of pressure insoles and IMUs are quasi-cyclic and varied with gait phases. To decrease variances of feature values within class and improve recognition performance, a two-level recognition strategy was utilized (Figure 3). First of all, the gait cycle was segmented into several continuous phases which could be detected with sensory signals; each phase had a corresponding classifier. It is worth emphasizing that the phases should be detected reliably for all the locomotion modes. In the first level of recognition, current gait phase was determined using sensory signals. And in the second level, the recognition result was obtained with the classifier of current gait phase. In our previous research [40], four nonadjacent phases with predefined sizes were defined, which did not meet the requirement for continuous recognition. In this study, we solved the problem by dividing the gait cycle into four adjacent phases: initial double-limb stance (DS1), single-limb stance (SS), terminal double-limb stance (DS2) and swing (SW). They could be detected with signals measured from both foot pressure insoles. For analysis windows containing signals of two adjacent phases, if more than half of the data belong to the first phase, the analysis window was labeled as the former phase; otherwise it was labeled as the latter phase. Six time-domain feature values (maximum, minimum, mean value, waveform length [41], standard deviation and root mean square) were calculated for signal channels of pressure insoles as well as IMUs. Feature values of the two kinds of sensor signals were combined together to generate the feature set (Figure 3). Linear discriminant analysis (LDA) classifier, which was similar as the one used in [42], was selected as the classifier for all the four phases.

#### 3.2.4. Post-Processing

Majority voting is a widely used post-processing approach [43], which utilizes classification results of multiple adjacent analysis windows to produce more accurate recognition decisions. However, potentials exist to improve this approach. In the original majority voting, each decision has the same weight value. This is not efficient when part of the decisions for voting are unreliable. We modified this method by adding weight value for each decision, which was determined by the posterior probability of the recognized decision with LDA classifier. Weight value of the *i*-th decision for majority voting was defined as

(1a), (1b) $$W_{i}=\{\begin{array}{ll} \frac{p_{i}-p_{0}}{1-p_{0}}, & p_{i}\geq p_{0},\\ 0, & p_{i}<p_{0}. \end{array}$$

where *p_i_* is the posterior probability of the *i*-th recognized decision (*i* = 1, 2,…, *N*), *p*_0_ is a threshold, and *N* is the number of decisions for majority voting.

Voting value of Mode-*j* was calculated by

(2) $$V_{j}=\sum_{i=1}^{N}W_{i}\cdot \eta_{ij},\;j=1,2,\ldots ,6$$

(3a), (3b) $$\eta_{ij}=\{\begin{array}{ll} 0, & R_{i}\neq M_{j},\\ 1, & R_{i}=M_{j}. \end{array}$$

where *R_i_* is the *i*-th recognized decision for voting and *M_j_* is Mode-*j*.

The final decision was determined by

(4a), (4b) $$D_{k}=\{\begin{array}{ll} D_{k-1}, & V_{\text{max}}=V_{s}\leq \gamma \cdot N,\\ M_{s}, & V_{\text{max}}=V_{s}>\gamma \cdot N. \end{array}$$

where *k* is the index of current decision, *γ* is a threshold and *M_s_* is the mode with the largest voting value.

In this research, the number of decisions for voting was *N* = 5, thresholds for weight value calculation and final decision determination were *p*_0_ = 0.5 and *γ* = 0.75, respectively.

### 3.3. Performance Evaluation

To make reasonable evaluations of recognition performance, we divided the locomotion period into steady locomotion periods (*i.e.*, no locomotion transitions) and locomotion transition periods. Locomotion transition periods were defined as follows. For transitions from stand to other locomotion modes, the locomotion transition period began when either foot left the ground and ended at the beginning of the next SS phase. For transitions from other locomotion modes to stand, the transition period began when the swing leg left the ground and ended when the swing leg contacted the ground before standing still. The transition period lasted for the length of a swing phase. For transitions between other locomotion modes, the transition period began when the swing leg left the ground just before transition and ended at the beginning of SS phase immediately after transition. The rest periods of the experiment trial were steady locomotion periods. Since the selection of the leading leg influenced the segmentation of locomotion transition periods, recognition performances of Set-A trials and Set-B trials should be evaluated separately. To reliably evaluate the overall performance of the system, we used leave-one-out-cross-validation (LOOCV) for result calculation and results of Set-A trials and Set-B trials were averaged.

#### 3.3.1. Steady Locomotion Periods

The average classification accuracy (CA) for steady locomotion periods was calculated by

(5) $$CA=\frac{N_{\text{cor}}}{N_{\text{total}}}\times 100\%$$

where *N_cor_* is the number of correctly recognized test events and *N_total_* is total number of test events in steady locomotion periods of the experiment trial.

As certain modes tended to be more frequently misclassified as some other modes, we constructed the confusion matrix to quantify the error distribution:

(6) $$C=\left(\begin{array}{llll} c_{11} & c_{12} & \cdots & c_{16}\\ c_{21} & c_{22} & \cdots & c_{26}\\ \cdots & \cdots & \cdots & \cdots \\ c_{61} & c_{62} & \cdots & c_{66} \end{array}\right)$$

where each element is defined as

(7) $$c_{ij}=\frac{n_{ij}}{{\bar{n}}_{i•}}\times 100\%.$$

*n_ij_* is the number of events in mode *i* that are mistakenly classified as mode *j* and *n̄*_*i*•_ is the total number of events in mode *i*. Thus, higher off-diagonal numbers indicate higher misclassification rates.

#### 3.3.2. Locomotion Transition Periods

To evaluate whether transition detections were made in time, the critical moment was defined. For transitions from other locomotion modes to stand, the critical moment was the time when the swing leg (either the measured leg or the unmeasured leg) contacted the ground before standing still. For the other locomotion transitions, the critical moment was the beginning of initial double stance (*i.e.*, foot-contact) for the measured foot. A correct locomotion transition detection should follow two rules. First, more than 30 consecutive correct recognition decisions were made. Second, after the first condition was satisfied, no false decisions occurred in the remaining transition period. The moment of the first correct decision was marked as *t_pre_*. Prediction time was calculated by

(8) $$T_{\text{pre}}=t_{c}-t_{\text{pre}}$$

where *t_c_* is the critical moment of the transition.

It is worth emphasizing that it's not a mistaken transition prediction when false recognitions were made, as long as they were corrected within the transition period. Considering prediction time as well as the number of missed detections, we calculated the adjusted prediction time (APD) to evaluate the performance of transition detection in the experiment trial. APD was defined as

(9) $$\text{APD}=\sum_{i=1}^{n}T_{\text{pre}}^{i}-T_{\text{max}}\cdot N_{\text{miss}}$$

where $T_{\text{pre}}^{i}$ is the prediction time of the *i*-th transition in the experiment trial, *n* is the number of transitions in the trial, *T_max_* is the time for punishing a missed detection, it was defined as 2000 ms in this research, *N_miss_* is the number of missed detections in the trial.

#### 3.3.3. Long-Time Use and Training Amounts

Apart from safety and powerful functions, long-time use and easy training are also important goals of developing a locomotion intent prediction system for prosthesis control. Complex training process and repeated training make it difficult for the user to accept the prosthesis. To evaluate the performance of our recognition system in long-time use, we used all the data measured in the first section of experiment for classifier training and tested the recognition performance with data measured some time (15 to 300 min) after training in the second section of experiment. Whether recognition performance deteriorated significantly over time can be illustrated by comparing average recognition accuracy and adjusted prediction time of each experiment pair. In addition, we used data of different number (1 to 20) of experiment pairs measured in the first section of the experiment to train the system and compared corresponding recognition results with data measured in the second section of the experiment. The necessary number of training pairs was determined when recognition results didn't improve significantly any more as the number of training pairs increased.

## 4. Results

### 4.1. Data for Classifier Training

Recognition accuracies in steady locomotion periods with different combinations of training and testing data were compared. We found classifiers trained with data of only Set-A trials or with data of only Set-B trials were insufficient to work appropriately for both situations (Table 1). When data of Set-A trials were used for classifier training, average testing accuracy of Set-A trials was 5.81% higher than that of Set-B trials. The difference was statically significant (*p* < 0.001, one-way repeated measures ANOVA). Similarly, testing accuracy of Set-B trials was 5.63% higher than that of Set-A trials when classifiers were trained with data of Set-B trials, and the difference was also statistically significant (*p* < 0.005). When classifiers were trained with data of Set-A trials and Set-B trials together, overall recognition accuracy was significantly higher than that when data of only Set-A trials (*p* < 0.001) or data of only Set-B trials (*p* < 0.005) were used for training. The results indicated that data of Set-A trials and Set-B trials should be used together for classifier training.

### 4.2. Influence of Window Size

**Figure 4.**
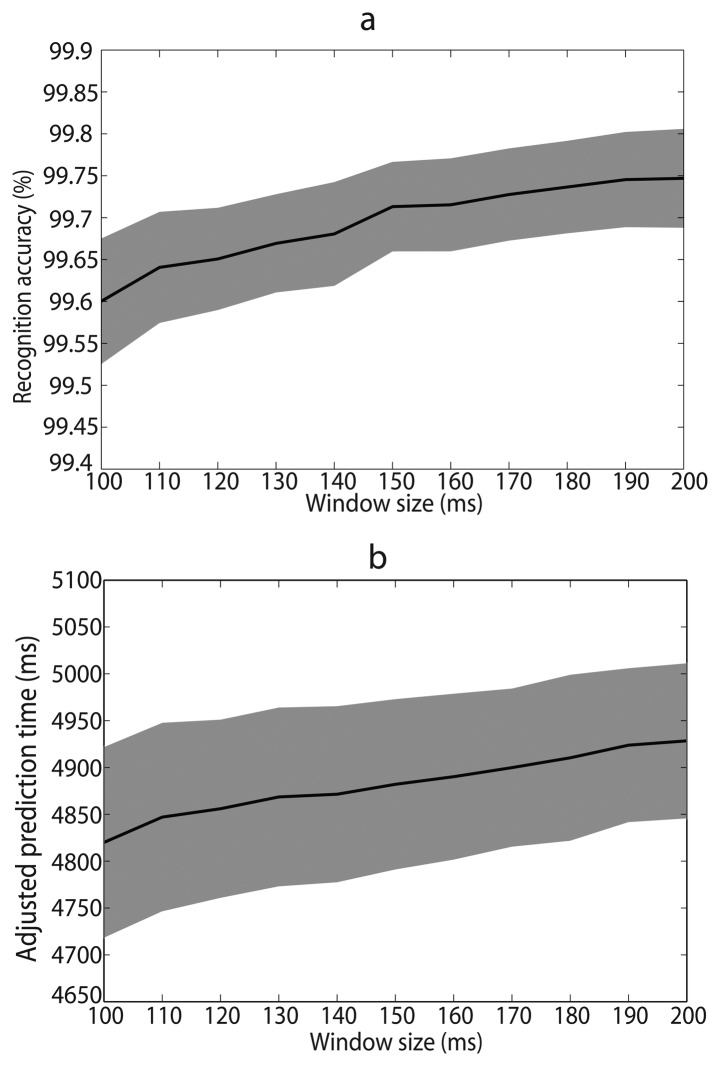
Average recognition accuracy (**a**) and adjusted prediction time (**b**) over seven able-bodied subjects with window size ranging from 100 ms to 200 ms. Color shades denote SEMs across subjects.

Window size influenced recognition accuracy in steady locomotion periods and prediction time in locomotion transition periods. The performances tended to be better as window size increased (Figure 4). However, larger analysis window increased computation complexity. Therefore, we should make a trade-off between recognition performance and computation complexity. One-way repeated measures ANOVAs were performed to analysis the influence of window size on recognition performance. Window size varied from 100 ms to 200 ms with 10 ms interval. The main effects were significant for both recognition accuracy and adjusted prediction time (*p* < 0.001). Pairwise comparisons between successive sizes yielded significant effect until 150 ms for recognition accuracy and 110 ms for adjusted prediction time. Therefore, we selected 150 ms as the optimal window size.

### 4.3. Modified Post-Processing Approach

**Figure 5.**
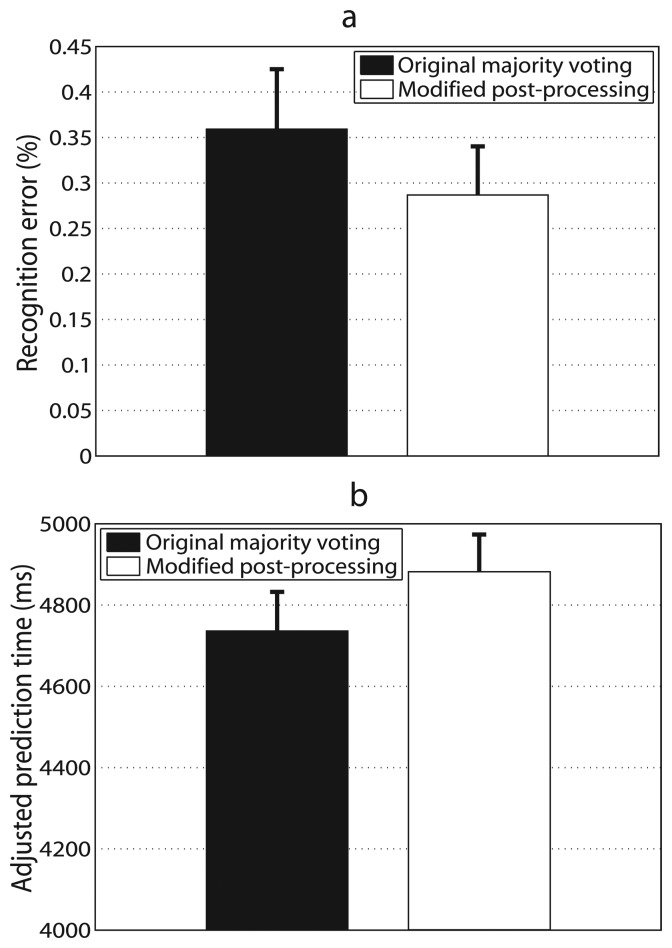
Average recognition error (**a**) and adjusted prediction time (**b**) over seven able-bodied subjects with the original majority voting and the modified post-processing approach. Error bars denote SEMs across subjects.

The modified post-processing approach performed better than the original majority voting (Figure 5). The recognition error decreased by 0.07% and the difference was statistically significant (*p* < 0.05). The adjusted prediction time increased by 146 ms and the difference was also statistically significant (*p* < 0.001). The number of decisions for the original majority voting was selected as 15, which could meet the requirement for recognition accuracy as well as prediction time. Results of both recognition errors and prediction time improved. Though recognition error decreased by only 0.07%, considering recognition errors for modified post-processing approach and the original majority voting were very low (0.29% and 0.36%, respectively), this decrease was relatively obvious. More importantly, these improvements were statically significant. It further demonstrated the modified post-processing approach was more efficient than the original majority voting.

### 4.4. Recognition Accuracy in Steady Locomotion Periods

Satisfactory recognition accuracy was obtained in steady locomotion periods (Table 2). Average recognition accuracies were 99.50% ± 0.15%, 99.49% ± 0.12%, 99.92% ± 0.04% and 99.59% ± 0.13% for DS1, SS, DS2 and SW, respectively. Most of the recognition errors were caused by the confusion of stair ascent and ramp ascent in DS1 and SW, walk misclassified as ramp descent in DS1 and stair ascent misclassified as walk in SS. The lowest recognition accuracy occurred in the recognition of ramp ascent. However, the accuracy was still as high as 98.90%. It indicated that the system could perform excellently for all the locomotion modes during the whole gait cycle.

### 4.5. Recognition Performance in Locomotion Transition Periods

Average prediction times and numbers of missed detections of every transition in the experiment trial were calculated (Table 3). Obvious differences of transition detection results between Set-A trials and Set-B trials were observed. As mentioned in Methods, the measured leg performed as different roles for transitions in Set-A trials and Set-B trials. The prediction time of transition was much larger with the unmeasured leg leading than that with the measured leg leading. Though detections for transitions of W→RD and RD→W in Set-A trials, during which the measured leg first transited to new terrains, could not be made before critical moments reliably, the other transitions could all be detected before critical moments. In addition, no missed detections was observed for all the trials of the seven able-bodied subjects.

### 4.6. Performance of Long-time Use

**Figure 6.**
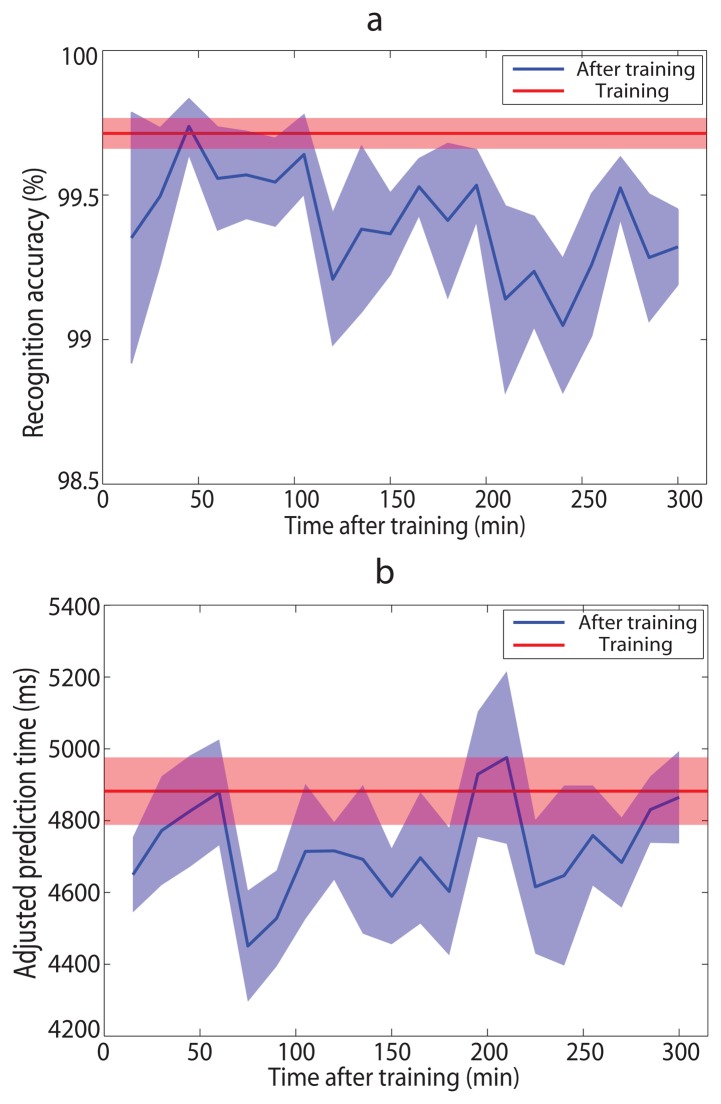
Average recognition accuracy (**a**) and adjusted prediction time (**b**) over seven able-bodied subjects with some time ranging from 15 min to 300 min after training. Color shades denote SEMs across subjects.

We evaluated the performance of the intent prediction system when it worked for a long time (up to 5 h) without retraining (Figure 6). The classifiers were trained with the data measured in the first section of the experiment. We used LOOCV to calculate training results of the system (recognition accuracy was 99.71% and adjusted prediction time was 4882 ms), as red lines shown in Figure 6. Average recognition accuracies over the seven subjects varied from 99.05% to 99.74% and average adjusted prediction times varied from 4451 ms to 4976 ms after training, as blue curves shown in Figure 6. Though overall recognition performances after training were a little worse than those of training results, they were still acceptable. In addition, deteriorating tendencies were not observed, unlike the EMG based recognition algorithms.

### 4.7. Influence of Training Amounts

**Figure 7.**
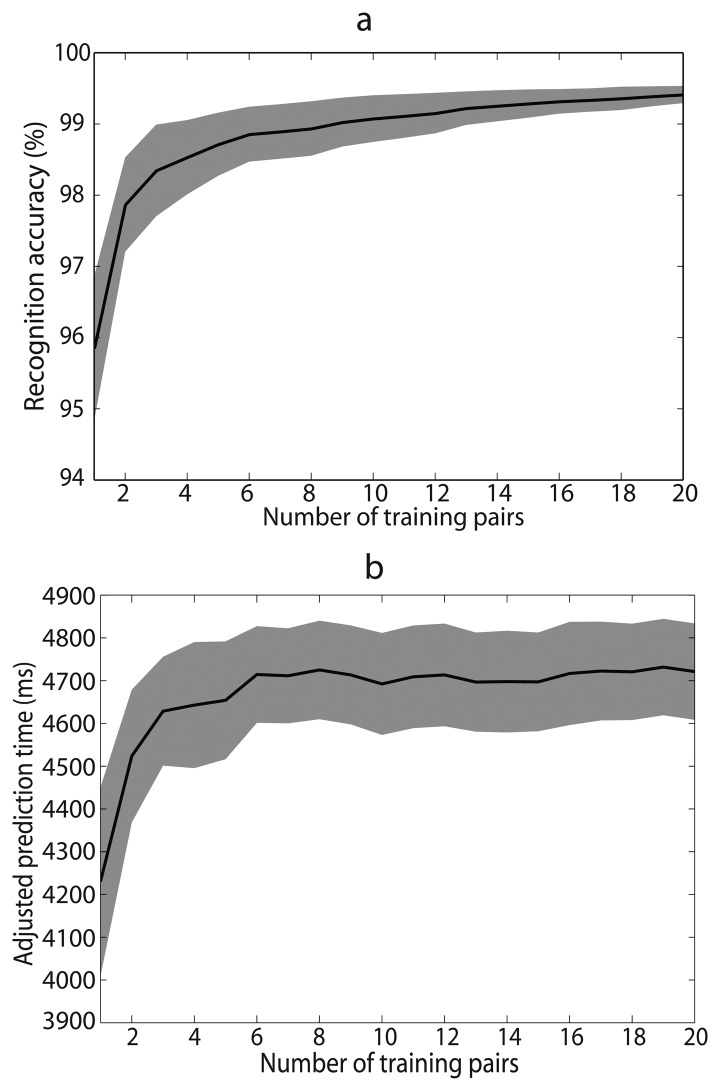
Average recognition accuracy (**a**) and adjusted prediction time (**b**) over seven able-bodied subjects with the number of training pairs ranging from 1 to 20. Color shades denote SEMs across subjects.

Recognition performances with different numbers of training pairs were compared (Figure 7). To avoid the same data being used for training as well as testing, we used the data measured in the first section of the experiment for classifier training and all the data measured in the second section for testing. As we could see from Figure 7, satisfactory recognition performance could be obtained with small number of training pairs, and the performance didn't improve significantly as training amounts increased. We performed one-way repeated measures ANOVAs to analysis the influence of training amounts. Pairwise comparisons between successive numbers yielded significant effect until 3 for recognition accuracy and 2 for prediction time. However, recognition accuracy still increased by more than 0.1% as training amounts increased until the number of training pairs reached 6. Therefore, we concluded that data of more than 6 experiment pairs could meet the requirement for classifier training.

### 4.8. Online Test

**Figure 8.**
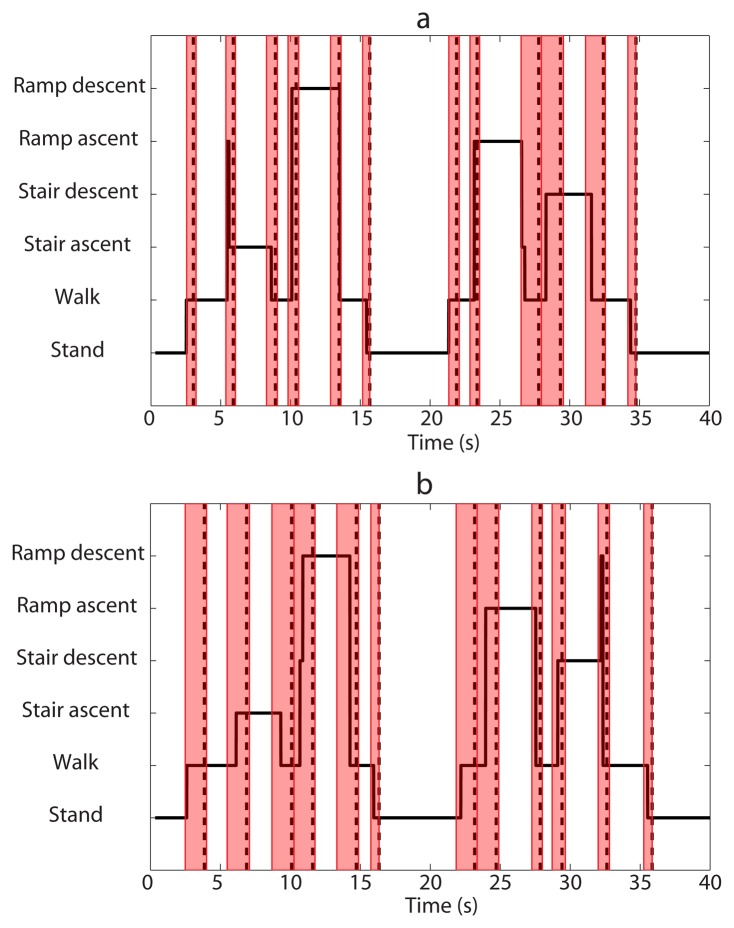
Recognition performances of online test experiments for Set-A experiment trial (**a**) and Set-B experiment trial (**b**). Data of 6 experiment pairs were used to train the system. The solid line denotes decision stream made by the locomotion intent recognition system. The dash line denotes the critical moment for each locomotion transition. Color shades denote locomotion transition periods of each trial.

To further validate the performance of the locomotion intent prediction system, we also tested it online. Recognition results of Set-A experiment trial and Set-B experiment trial were shown in Figure 8, respectively. The system was trained with data of only 6 experiment pairs and performed well for most of the experiment periods. For the Set-A experiment trial, false recognitions occurred during transitions of W→SA and RA→W, which were caused by the confusion of stair ascent and ramp ascent. However, the periods of false recognition were very short, and recognition stream soon turned to be correct before critical times for transitions. Transition of RD→W was detected 60 ms after critical moment, and the other transitions were predicted in advance. For the Set-B experiment trial, false recognitions occurred during transitions of W→RD and SD→W, which were caused by the confusion of stair descent and ramp descent. But the periods of false recognition were also very short, and recognition stream soon turned to be correct before critical times for transitions. All transitions were successfully predicted in advance. The overall online test performances were in consistence with the offline evaluation results.

Note that a video is included in the supplemental material, which recorded online test experiments of a Set-A trial and a Set-B trial.

## 5. Discussion

We developed a promising locomotion intent prediction system for the control of powered lower-limb prostheses. With the fusion of IMU signals and foot pressure signals at the feature level, the system realized real-time continuous recognition of locomotion modes with high accuracy and could even detect transitions between different locomotion modes in advance for most of the transitions. In addition, with small amount of training data, the system could perform satisfactory and reliable work for a long time without retraining. These results demonstrated the prospect of realizing safe and fluent control for powered lower-limb prostheses with this locomotion intent prediction system.

The locomotion intent prediction system is promising to improve the performance of powered lower-limb prosthesis for the following reasons. First, the system performs well for frequently occurred locomotion modes and locomotion transitions in daily life. To evaluate whether this system can meet the requirement of daily activities, we considered more locomotion modes and locomotion transitions than previous studies [14,15,18]. More importantly, overall recognition accuracy (99.71%) and the performance of transition detection (no missed detection) are excellent. For most of the locomotion transitions, the system can detect them in advance, which leaves more time for the powered prosthesis to react to terrain changes. Though transitions of W→RD and RD→W in Set-A trials could not be detected before critical moments reliably, the impacts on powered prosthesis control are not significant, because control performance also depends on other factors such as mechanical property and control strategy of the powered prosthesis. Some powered lower-limb prostheses tend to be passive for a period of time after foot contact (*i.e.*, the critical moment), during which no motor control is needed [1,2,11]. Therefore, transition detections with small time delays can be accepted.

Second, the system can meet the requirement of naturally selection of leading leg for locomotion transitions. The goal of powered lower-limb prosthesis research is enabling amputees to walk with normal gaits. Therefore, the system is designed with the same consideration. In the experiment, two legs should naturally alternate during walking in different terrains and recognition performances of both Set-A and Set-B trials should be acceptable. We used data of Set-A trials and data of Set-B trials together for classifier training. Recognition accuracies in steady locomotion periods are above 99% for both Set-A and Set-B trials (Table 1). However, as less time is left for transition detection when the measured leg first transited to a new locomotion mode, it is more difficult to make an accurate detection (Table 3). We report transition detection results of both Set-A and Set-B trials, and validate they are acceptable for both situations.

Third, the system is easy trained and capable of long-time use without retraining. As we can see from Figure 7, data of 6 or more experiment pairs (*i.e.*, 12 experiment trials) are enough to train a reliable system. In this research, the measured time of each experiment trial is 40 s. Therefore, the training process only takes about 15 min, including data processing and rest time. More importantly, the performance of the system is close to the training results even after five hours (Figure 6). Decaying tendency of recognition performance which occurred in EMG based recognition systems [21] was not observed. Thus, amputees don't need to worry about cumbersome system training and possible frequent retraining.

Additionally, the system is easy to integrate with a powered lower-limb prosthesis in hardware as well as in control strategy. To our knowledge, some previous studies have implemented recognition algorithms in embedded systems [44,45]. And as signals measured from IMUs have clear physical meanings, they have already been integrated with some prosthetic systems and used for prosthesis control [10,46,47]. In addition, some of the existing powered lower-limb prostheses are controlled with finite-state-machine [1,2,7,11]. But most of them required users to manually switch the control mode, which is inconvenient for practical application. The proposed recognition system can be used to detect current locomotion mode, and automatically select the appropriate control mode.

Our intent prediction system show great potential for powered lower-limb prosthesis control. However, deficiencies and improvement spaces exist for the two-level recognition strategy. First of all, gait phases are determined using foot-contact information of both feet. However, it is cumbersome to wear an extra pressure insole on the unmeasured side, which may be a limitation for clinical applications. We will find new ways to segment the gait cycle with sensor signals measured from only the measured leg by adding extra sensors, such as a load cell integrated with the prosthesis [48]. Second, it is not necessary for all the phases to use the same classifier, feature set, window size and other parameters for classification and post-processing, because signals of different phases have their own characteristics. For example, IMU signals vary a lot in SW phase, while has relatively small variance in SS phase. We will perform parameter optimization individually for each phase, and it could make the locomotion intent prediction system more efficient. In the future, we will test the system on amputee subjects with powered lower-limb prostheses worn and evaluate the performance of prosthesis control.

## Figures and Tables

**Table 1.** Recognition Accuracy (Mean ± SEM) with Different Combinations of Training and Testing Data (%).

	**Testing**		
**Training**	**Set-A**	**Set-B**	**Set-A & Set-B**
Set-A	99.26 ± 0.21	93.45 ± 0.68	96.35 ± 0.38
Set-B	93.61 ± 1.26	99.24 ± 0.36	96.43 ± 0.76
Set-A & Set-B	99.37 ± 0.14	99.64 ± 0.19	99.51 ± 0.13

**Table 2.** Confusion Matrix (Mean ± SEM) for Seven Able-bodied Subjects in Steady Locomotion Periods (%).

		**Estimated Mode**					

**Phase**	**Targeted Mode**	**Stand**	**Walk**	**Stair Ascent**	**Stair Descent**	**Ramp Ascent**	**Ramp Descent**
DS1	Stand	99.96 ± 0.03	0.00 ± 0.00	0.00 0.00	0.00 ± 0.00	0.03 ± 0.03	0.00 ± 0.00
	Walk	0.00 ± 0.00	98.99 ± 0.29	0.00 ± 0.00	0.20 ± 0.13	0.02 ± 0.02	0.78 ± 0.27
	Stair Ascent	0.00 ± 0.00	0.00 ± 0.00	99.53 ± 0.18	0.00 ± 0.00	0.47 ± 0.18	0.00 ± 0.00
	Stair Descent	0.10 ± 0.10	0.30 ± 0.21	0.00 ± 0.00	99.50 ± 0.24	0.00 ± 0.00	0.09 ± 0.07
	Ramp Ascent	0.00 ± 0.00	0.01 ± 0.01	0.84 ± 0.24	0.00 ± 0.00	99.15 ± 0.18	0.00 ± 0.00
	Ramp Descent	0.00 ± 0.00	0.10 ± 0.05	0.00 ± 0.00	0.00 ± 0.00	0.00 ± 0.00	99.90 ± 0.05

SS	Stand	99.76 ± 0.07	0.24 ± 0.07	0.00 ± 0.00	0.00 ± 0.00	0.00 ± 0.00	0.00 ± 0.00
	Walk	0.22 ± 0.19	99.35 ± 0.16	0.15 ± 0.05	0.24 ± 0.05	0.00 ± 0.00	0.04 ± 0.02
	Stair Ascent	0.23 ± 0.23	0.61 ± 0.37	99.13 ± 0.40	0.02 ± 0.02	0.00 ± 0.00	0.00 ± 0.00
	Stair Descent	0.38 ± 0.30	0.25 ± 0.17	0.02 ± 0.02	99.17 ± 0.49	0.00 ± 0.00	0.18 ± 0.18
	Ramp Ascent	0.19 ± 0.19	0.06 ± 0.04	0.00 ± 0.00	0.00 ± 0.00	99.75 ± 0.23	0.00 ± 0.00
	Ramp Descent	0.20 ± 0.20	0.01 ± 0.00	0.00 ± 0.00	0.00 ± 0.00	0.00 ± 0.00	99.79 ± 0.20

DS2	Stand	99.97 ± 0.03	0.03 ± 0.03	0.00 ± 0.00	0.00 ± 0.00	0.00 ± 0.00	0.00 ± 0.00
	Walk	0.00 ± 0.00	99.92 ± 0.06	0.08 ± 0.06	0.00 ± 0.00	0.00 ± 0.00	0.00 ± 0.00
	Stair Ascent	0.00 ± 0.00	0.11 ± 0.07	99.89 ± 0.07	0.00 ± 0.00	0.00 ± 0.00	0.00 ± 0.00
	Stair Descent	0.00 ± 0.00	0.00 ± 0.00	0.00 ± 0.00	100.00 ± 0.00	0.00 ± 0.00	0.00 ± 0.00
	Ramp Ascent	0.00 ± 0.00	0.06 ± 0.04	0.14 ± 0.09	0.06 ± 0.06	99.74 ± 0.15	0.00 ± 0.00
	Ramp Descent	0.00 ± 0.00	0.00 ± 0.00	0.00 ± 0.00	0.00 ± 0.00	0.00 ± 0.00	100.00 ± 0.00

SW	Stand	99.87 ± 0.05	0.13 ± 0.05	0.00 ± 0.00	0.00 ± 0.00	0.00 ± 0.00	0.00 ± 0.00
	Walk	0.00 ± 0.00	99.81 ± 0.12	0.00 ± 0.00	0.00 ± 0.00	0.00 ± 0.00	0.19 ± 0.12
	Stair Ascent	0.00 ± 0.00	0.00 ± 0.00	99.75 ± 0.12	0.00 ± 0.00	0.25 ± 0.12	0.00 ± 0.00
	Stair Descent	0.00 ± 0.00	0.17 ± 0.11	0.00 ± 0.00	99.60 ± 0.21	0.16 ± 0.11	0.07 ± 0.05
	Ramp Ascent	0.00 ± 0.00	0.00 ± 0.00	1.10 ± 0.34	0.00 ± 0.00	98.90 ± 0.34	0.00 ± 0.00
	Ramp Descent	0.00 ± 0.00	0.18 ± 0.18	0.00 ± 0.00	0.21 ± 0.11	0.00 ± 0.00	99.61 ± 0.18

**Table 3.** Performance of Transition Detection (Mean ± SEM) for Seven Able-bodied Subjects in Locomotion Transition Periods.

	**Set-A Trials**		**Set-B Trials**	
**Transitions**	**Prediction Time (ms)**	**Number of Missed Detections**	**Prediction Time (ms)**	**Number of Missed Detections**
S→W	451 ± 10	0	1035 ± 21	0
W→AS	222 ± 14	0	628 ± 20	0
AS→W	204 ± 9	0	732 ± 29	0
W→DR	27 ± 33	0	508 ± 19	0
DR→W	−120 ± 13	0	284 ± 35	0
W→S	256 ± 19	0	110 ± 16	0
S→W	396 ± 17	0	891 ± 21	0
W→AR	116 ± 17	0	447 ± 19	0
AR→W	822 ± 28	0	270 ± 13	0
W→DS	887 ± 34	0	210 ± 14	0
DS→W	886 ± 37	0	117 ± 13	0
W→S	241 ± 23	0	145 ± 17	0

Note: A negative value of prediction time indicates the decision is made after the critical moment.
